# Review of Cyanotoxicity Studies Based on Cell Cultures

**DOI:** 10.1155/2022/5647178

**Published:** 2022-04-25

**Authors:** Iliyana Sazdova, Milena Keremidarska-Markova, Mariela Chichova, Blagoy Uzunov, Georgi Nikolaev, Mitko Mladenov, Rudolf Schubert, Maya Stoyneva-Gärtner, Hristo S. Gagov

**Affiliations:** ^1^Department of Animal and Human Physiology, Faculty of Biology, St. Kliment Ohridski University of Sofia, 8 Dragan Tsankov Blvd., Sofia 1164, Bulgaria; ^2^Department of Botany, Faculty of Biology, St. Kliment Ohridski University of Sofia, 8 Dragan Tsankov Blvd. 1164 Sofia, Bulgaria; ^3^Department of Cytology, Histology and Embryology, Faculty of Biology, St. Kliment Ohridski University of Sofia, 8 Dragan Tsankov Blvd, Sofia 1164, Bulgaria; ^4^Faculty of Natural Sciences and Mathematics, Institute of Biology, Sts, Cyril and Methodius University, Skopje 1000, North Macedonia; ^5^Department of Physiology, Institute of Theoretical Medicine, Medical Faculty, University of Augsburg, Augsburg 86159, Germany

## Abstract

Cyanotoxins (CTs) are a large and diverse group of toxins produced by the peculiar photosynthetic prokaryotes of the domain Cyanoprokaryota. Toxin-producing aquatic cyanoprokaryotes can develop in mass, causing “water blooms” or “cyanoblooms,” which may lead to environmental disaster—water poisoning, extinction of aquatic life, and even to human death. CT studies on single cells and cells in culture are an important stage of toxicological studies with increasing impact for their further use for scientific and clinical purposes, and for policies of environmental protection. The higher cost of animal use and continuous resistance to the use of animals for scientific and toxicological studies lead to a progressive increase of cell lines use. This review aims to present (1) the important results of the effects of CT on human and animal cell lines, (2) the methods and concentrations used to obtain these results, (3) the studied cell lines and their tissues of origin, and (4) the intracellular targets of CT. CTs reviewed are presented in alphabetical order as follows: aeruginosins, anatoxins, BMAA (*β*-N-methylamino-L-alanine), cylindrospermopsins, depsipeptides, lipopolysaccharides, lyngbyatoxins, microcystins, nodularins, cyanobacterial retinoids, and saxitoxins. The presence of all these data in a review allows in one look to advance the research on CT using cell cultures by facilitating the selection of the most appropriate methods, conditions, and cell lines for future toxicological, pharmacological, and physiological studies.

## 1. Introduction

Cyanotoxins (CTs) are a large and diverse group of toxins produced by the peculiar photosynthetic prokaryotes of the domain Eubacteria, commonly known as cyanobacteria or blue-green algae, and since 1999 named Cyanoprokaryota [1, 2]. Some aquatic cyanoprokaryotes can develop in mass, causing so-called “water blooms” or “cyanoblooms” [3]. When such blooms are formed by toxin-producing cyanoprokaryotic algae, they are considered harmful and are usually abbreviated as CyanoHABs. The toxic substances are transported through the food webs and may reach people and animals by drinking water, or through other exposure routes, which include recreational activities or consumption of so-called “sea-food”, which includes both freshwater and marine organisms [3–5]. The excretion of toxic compounds may lead to environmental disasters—water poisoning, extinction of aquatic life, and even to human death [3–5]. Current climate changes and anthropogenic press can intensify and increase the frequency of these hazardous ecological events [3, 6]. Although most research addresses aquatic toxin producers, there is a growing body of evidence on such producers from aeroterrestrial and extreme habitats, and among airborne algae as well, with a considerable number of detected toxins and outlining of additional exposure route through consumption of crops, which have been irrigated by contaminated water [7, 8].

Different approaches have been applied to classify CT, two of which are the most common: by the target of their action, or by chemical composition. By target, CT are classified as hepatotoxins, neurotoxins, dermatoxins, and cytotoxins, whereas chemically they are divided in peptides, alkaloids, phosphorylated cyclic N-hydroxyguanine, diaminoacids, and lipopolysaccharides, the last widely recognized as endotoxins. Prolonged use of drinking water, contaminated with low-doses CTs, may have also carcinogenic effect [6]. Thus, microcystin-LR (MC-LR), the most toxic MC, is considered to express tumor promoting effect mainly by violating phosphorylation-dependent regulations of cellular proteins [new 9 Brozman et al., 2020]. The pleiotropic downstream mechanisms link MC-LR-dependent inhibition of eucaryotic protein phosphatases (PPs) PP1, PP2A, phospho-PP4, and phospho-PP5 [2] to tumor promotion and neoplastic transformation by cell growth induction, reactive oxygen species (ROS) generation, oxidative stress, mitochondrial DNA impairment, and by the transformation of cell phenotype [9]. Chronic proinflammatory effect of MC-LR alone or a combination with another CT-like cylindrospermopsin (CYN) may additionally stimulate the neoplastic transformation and tumor progression [6, 10].

Cell cultures are very convenient for toxicological studies. They allow to reveal the mechanisms of cytotoxic effects, the affected tissues, intracellular targets, and ways to minimize cytotoxicity [11]. The use of human cell lines in toxicological studies is a fast and effective way to investigate the damaging effects of toxins in humans and to identify the most sensitive tissues.

Although different methods are developed for testing of toxins in cell- and animal-based studies, during the last years, the trials on the use of animals have significantly decreased. This is caused by the high cost of these types of clinical trials and increasing resistance to the use of animals for scientific studies. Therefore, the significance and use of cell lines is gradually increasing.

This review aims to present (1) the important results of the effects of CT on human and animal cell lines; (2) the methods and concentrations used to obtain these results, (3) the studied cell lines, and (4) the intracellular targets of CT. The presence of all these data in a review allows in one look to advance the toxicological and pharmacological studies of CT using cell cultures by facilitating the selection of the most appropriate methods, conditions, and cell lines.

## 2. Cyanotoxicity on Cell and Cell Cultures

### 2.1. Cytotoxicity of Aeruginosins (Table 1)

Aeruginosin CT contains as a basic structure 2-carboxy-6-hydroxyoctahydroindol that are serine protease inhibitors [12]. They inhibit trypsin-like serine proteases and for this activity are important in the search for new anticoagulants [13].

### 2.2. Cytotoxicity of Anatoxins (Table 2)

Anatoxins-a are two types of low molecular bicyclic amino alkaloids: anatoxin-a (ANTX) and homoanatoxin-a (hANTX). The best known of them is ANTX, which was the first to be identified as a low molecular alkaloid (165 Da). hANTX is a homologue of anatoxin-a with molecular weight 179 Da and has propionyl instead of an acetyl group at C-2. ANTX and anatoxin-a (S) (ANTX(S)) are neurotoxins. ANTX binds competitively to acetylcholine receptors, while anatoxin-a (S) inhibits acetylcholine esterase [2].

### 2.3. Cytotoxicity of BMAA (Table 3)

*β*-N-methylamino-L-alanine (BMAA) is an environmental nonprotein and toxic amino acid that may harm nervous system via oxidative stress, binding to neuromelanin, forming high toxic metabolites like formaldehyde or inhibiting enzyme activity of glutathione reductase, *β*-amilase, catalase, and RNase H, and in this way to provoke sporadic neurodegenerative development, such as Alzheimer's disease and amyotrophic lateral sclerosis [20, 27, 28]. In addition, BMAA generates a carbamate, which is neurotoxin because it acts as ionotropic and metabotropic glutamate receptors agonist [21] and references therein.

### 2.4. Cytotoxicity of CYN (Table 4)

CYN is a cyclic quinidine alkaloid combined with hydroxymethyl uracil [49]. It has two epimers, which are equally toxic and are differentiated by the hydroxyl bridge CYN and 7-epi-CYN, and an additional variant 7-deoxy-CYN occurs in natural waters [49]. CYN has been classified mainly as hepatotoxin, but it has also neurotoxic and genotoxic effects and inhibits protein synthesis [3]. It targets kidneys, lungs, heart, spleen, eyes, ovaries, T-cells, neutrophils, and vascular endothelium [50]. CYN may induce oxidative stress, decrease cell viability, and damage mitochondria (discussed by Chichova et al. [35]).

### 2.5. Cytotoxicity of Depsipeptides (Table 5)

Depsipeptides are palmyramide A (Pal A), apratoxin *D* (AT D), coibamide A (CoA), ichthyopeptins A (Ich A) and B (Ich B), kahalalide F (KF), 4-Fluoro-3-methyl-benzylamino-KF (KF2), morpholin-4-yl-benzylamino-KF (KF4), homodolastatin 16 (HD16), lagunamide C–Lag C, pitipeptolides–Pit A-F, aurilides and wewakpeptins A-D. Depsipeptides show cytotoxic activity and are protease inhibitors selective for chymotrypsin, leukocyte, and pancreatic elastases. They negatively influence the metabolism of human astrocytes [63].

### 2.6. Cytotoxicity of Lipopolysaccharides (LPS, Table 6)

LPS consist of lipid A, the core polysaccharides (mainly glucosamine) and an outer polysaccharide chain, and are common compounds of the cell walls of cyanoprokaryotes and Gram-negative bacteria [49]. They have an inflammatory effect and promote cytokine secretion [3].

### 2.7. Cytotoxicity of Lyngbyatoxins (Table 7)

Lyngbyatoxins were first identified from Moorea producens (formerly Lyngbya majuscula). They are tumor-promoting agents which bound eucaryotic protein kinase C (PKC) isozymes [3].

### 2.8. Cytotoxicity of MCs (Table 8)

MC are cyclic nonribosomal heptapeptides with low molecular weight (800–1100 Da), which contain several uncommon nonproteinogenic amino acids such as N-methyldehydroalanin (MDHA) derivatives and the uncommon *β*-amino acid 3-amino-9-methoxy-2,6,8-trimethyldeca-4,6-dienoic acid (ADDA). MC are lipophilic toxins very resistant to hydrolysis, oxidation, and high temperatures. The main route of human exposure is the ingestion of contaminated drinking water, consumption of contaminated food or algal dietary supplements, and body contact, while more occasional routes are hemodialysis and inhalation. MC are classified mainly as hepatotoxins because they block eucaryotic PP (PP1, 2A and phosphoprotein phosphatases PPP4, PPP5) [2] through irreversible covalent binding [97]. Chronic and subchronic exposure to MC seems to be tumor promoting because they can increase the incidence of hepatic tumors in humans. MC could also enhance the oxidative stress. Additional target of MC in high concentrations is the ß-subunit of ATP synthase, causing mitochondrial apoptotic signaling. MC have hepatotoxic and tumor promoting action [3].

### 2.9. Cytotoxicity of Nodularins (Table 9)

Nodularins (NODs) are cyclic nonribosomal pentapeptides and contain several unusual nonproteinogenic amino acids such as N-methyl-didehydroaminobutyric acid and the *ßβ*-amino acid (all-S, all-E)-3-amino-9-methoxy-2,6,8-trimethyl-10-phenyldeca-4,6-dienoic acid (ADDA). Ten variants have been discovered with nodularin-R being the predominant toxin variant. NODs are relatively stable compounds, with low sensitivity to light or temperature. NOD affects hepatocytes binding their PPs by noncovalent bonds, which increases the rate of phosphorylation. They are often attributed to gastroenteritis, allergic irritation reactions, and liver diseases. Nodularin-R is the most notorious as a potent hepatotoxin that may cause serious damage to the liver of humans and other animals. NODs have similar effects as microcystins and weak carcinogenicity [3].

### 2.10. Cytotoxicity of Retinoids from Cyanobacteria (Table 10)

Retinol, a novel retinoic acid (RA) analogue 7-hydroxy RA, 4-oxo-RA, and several analogues were identified in cyanobacterial blooms [110]. They act as RA receptors that may cause different malformations, as well as to have a teratogenic effect on aqueous animals.

### 2.11. Cytotoxicity of saxitoxins (Table 11)

Saxitoxin (SXT) is a collective name for a group of more than 20 cyclic nonribosomal peptide molecules, formed by sulphation at different sites of two basic molecules: SXT and neo-SXT. Based on their toxicology, SXT are grouped in three classes—carbamate derivatives, gonyautoxins, N-sulfocarbomoyl derivatives, and decarbomoyl derivatives—decarbamoylsaxitoxin. They have a neurotoxic effect by blocking voltage-gated sodium channels [3].

## 3. Limitations

Studies on cell cultures cannot reveal all possible effects of toxins on the human body. This is due to the following reasons: (1) no matter how many cultures are tested, they will not cover the whole variety of cells in the body; (2) there are often significant differences between the cells in culture, the primary cell lines and the cells in the body tissues in the quantity and quality of expressed proteins (genes expression), metabolic pathways and cell function [113–115]. Therefore, results from cells in culture cannot be directly transferred to the tissue of origin or of which they will form. (3) Numerous regulations are active continuously and simultaneously in the organism, and their cross-influence cannot be simulated in experiments with cell cultures. (4) Parameters like LC_50_ or ID_50_ are different for cells in culture and human body.

## 4. Perspectives

The use of cell cultures in toxicological studies will remain the main approach due to its speed, relatively low cost, reproducibility, precision with respect to the studied intracellular components, and ethical acceptability. The use of cell cocultures [116–118] and *in vitro* formed organ-like structures such as artificial neuronal network [119], cardiomyocyte spheroids with contractile activity [120], and organ-on-a-chip systems [121], which are functionally closer to the human body [11], will increase in the future.

## 5. Conclusion

The presence of all these data on the cytotoxicity of aeruginosins, anatoxins, cylindrospermopsin, depsipeptides, lipopolysaccharides, lyngbyatoxins, microcystins, nodularins, cyanobacterial retinoids, and saxitoxins in a review is a great advantage. It allows the advancement of research on CT using cell cultures by facilitating the selection of the most appropriate methods, conditions, and cell lines for toxicological and pharmacological studies. In addition, it could increase the use of CT in functional studies of their intracellular targets. Therefore, this review allows in one look to advance the toxicological, physiological, and pharmacological studies of CT by the knowledge of their harmful effects with a focus on human and animal health as well as on environmental protection.

## Figures and Tables

**Table 1:** Cytotoxicity of aeruginosins.

Cell type	Assay	Conditions	Tissue of origin	Main effects	Targets	Ref.
Huh7 cells	EROD assay, treatment with TNF-*α*	Aeruginosin-865A 50 and 100 µmol/L	Human hepatoma cell line	Anti-inflammatory activity by inhibition of IL-8 and TNF-*α* expression; induce expression of cytochrome P_450_ 1A (CYP1A)	DNA	[12]
HLMVEC	IL-8 and ICAM-1 assay upon stimulation with human tumor necrosis factor *α* (hTNF-*α*)	Aeruginosin-865 0.1–100 µg/mL/18 h of 0.1 ngm/L hTNF-*α* stimulated cells	Human lung microvascular endothelial cells	Anti-inflammatory activity by down-regulation of IL-8 (EC_50_ : 4.0 ± 1.7 mM) and intercellular adhesion molecule 1 (ICAM-1; 57.8 ± 15.5 mM)	Inhibits NF-kappa B translocation to the nucleus	[13]
WEHI-13VAR	Lactate dehydrogenase (LDH) cytotoxicity assay	Aeruginosin-865 10–200 µM	Mouse fibrosarcoma cells	Cytotoxic effect of aeruginosin-865 at 200 µM only		[14]

Abbreviations: EROD – ethoxyresorufin-O-deethylase; hTNF*α* – human tumor necrosis factor *α*; ICAM-1 – intercellular adhesion molecule-1; IL-8 – interleukin 8; TNF-*α* – tumor necrosis factor *aα*.

**Table 2:** Cytotoxicity of ANTX, hANTX and ANTX(S).

Cell type	Assay	Conditions	Tissue of origin	Main effects	Targets	Ref.
RAW 264.7, BV-2, N2a	MTT assay, caspase-glo 3/7 assay, ELISA, TNF-*α* measurement	MC-LR, CYN, ANTX-a	Murine macrophage-like RAW264.7, immortalised microglial BV-2, neuroblastoma N2a cell lines	CYN, MC-LR and ANTX in a mixture are 3–15 times more potent at inducing apoptosis and inflammation	TNF-*α*	[15]
Oocytes, M10 cells	Patch-clamp, ^86^Rb ^+^ influx	ANTX	*Xenopus* oocytes, human hepatoma cell line	*α*7-nAChR agonist with EC_50_ = 0.58 *μ*M (nicotinic current in oocytes), *α*4*β*2-nAChR EC_50_ : 48 n*M* by ^86^Rb ^+^ influx in M10 cells	*α*7-nAChR, *α*4*β*2-nAChR, Ach	[16, 17]
GH_4_C_1_	^45^Ca^2+^ influx, [^3^H]-ACh release,	hANTX water extract, 1–20 mg/mL	Rat anterior pituitary cell line	hANTX-activated voltage-gated Ca^2+^ channels and AChR release	Voltage-gated Ca^2+^ channels, AChR	[18]
Chromaffin cell culture	HPLC	ANTX 0.1–100 *μ*M	Bovine adrenal chromaffin cell culture	Catecholamine release activation above 0.3 *μ*M ANTX	Secretion of catecholamines	[19]

Abbreviations: Ach – acetylcholine; AChR – acetylcholine receptor; CYN – cylindrospermopsin; HPLC – high-performance liquid chromatography; MC-LR – microcystin-LR; MTT – 3-(4,5-dimethylthiazol-2-yl)-2,5-diphenyltetrazolium bromide; nAChR – nicotinic acetylcholine receptor.

**Table 3:** Cytotoxicity of BMAA.

Cell type	Assay	Conditions	Tissue of origin	Main effects	Ref.
HepG2 cells, Caco-2	Isotopically labelled amino acids; metabolic activity; apoptotic and necrotic assays		Human hepatocellular carcinoma and human colorectal epithelial adenocarcinoma cell line	BMAA did not affect the common proteinogenic amino acid metabolic pathways; in the presence of amino acids cellular uptake of BMAA is substantially reduced	[20]
SH-SY5Y	LDH assay; qPCR; Western Blot	L-BMAA 1 mM/17 h	Human neuroblastoma cells	Conversion of procaspase-3 (32 kDa) to active caspase-3 p17 and apoptosis	[21]
SH-SY5Y	LDH assay; qPCR; Western Blot	L-BMAA 1 mM/17 h and longer for 24–96 h	Human neuroblastoma cells	Misincorporation of L-BMAA protein aggregation, upregulation of lysosomal enzymes and apoptosis; proteolitic stress in prolonged exposure	[22]
SH-SY5Y		Low L-BMAA (≥0.1 mM)/48 h; high L-BMAA (≥2 mM)/48 h	Human neuroblastoma cells	Low L-BMAA increases protein ubiquitination, 20S proteasomal and caspase 12 activity, stress marker CHOP expression; enhances phosphorylation of elf2*α* in SH-SY5Y cells; high L-BMAA increases ROS and protein oxidization	[23]
OEC	LDH assay, MTS assay, Ca^2+^ influx assay, DCFDA assay for ROS, DNA damage assay	BMAA 0.1–3 mM/48 h	Rat olfactory ensheathing cells (special glial cells)	Cytotoxic, increases Ca^2+^ influx, and ROS production; disrupts mitochondrial activity	[24]
Primary neurons	LDH assay, MTS assay, Ca^2+^ influx assay, DCFDA assay for ROS, DNA damage assay	BMAA 0.1–1 mM/48 h	Primary neurons were obtained from 16 to 19 old foetuses and mixed brain cell cultures	BMAA increases Ca^2+^ influx and DNA damage, enhances production of ROS, disrupts activity of mitochondria	[25]
SH-SY5Y, HT22, Neuro-2a	MTT assay, siRNA transfection, flow cytometry for DNA content	BMAA 1–3 mM/12, 24, and 48 h	Human neuroblastoma cells; mouse hippocampal cell line; mouse neuroblastoma cell line	L-BMAA-induced ER-stress mediated apoptosis via upregulation of ER-stress sentinels, phosphorylation of JNK, p38 and ERK, CHOP activation	[26]
SH-SY5Y, MRC-5, HUVEC	Liquid chromatography tandem mass spectrometry, radiolabeled ^3^H-BMAA assay, LDH assay,	0.3 mM BMAA and 300 mM L-serine for 96 hours	Human neuroblastoma and human lung fibroblast cell line, human umbilical endothelial cells	BMAA is misincorporated in place of L-serine into human proteins and this is inhibited by L-serine	[22]

Abbreviations: CHOP – C/EBP homologous protein; DCFDA – 2′,7′-Dichlorofluorescin diacetate assay; ER – endoplasmic reticulum; JNK – c-Jun *N*-terminal kinase; MTS – 3-(4,5-dimethylthiazol-2-yl)-5-(3-carboxymethoxyphenyl)-2-(4-sulfophenyl)-2H-tetrazolium.

**Table 4:** Cytotoxicity of CYN.

Cell type	Assay	Conditions	Tissue of origin	Main effects	Targets	Ref.
CaCo-2	Neutral red uptake	1.1 mg/g dw; 0.08–1.25 mg dw/mL/48 h	Immortalized human colorectal adenocarcinoma cell line	Cytotoxicity, EC_50_ : 0.4 ± 0.1 mg dw/mL		[29]
CaCo-2	Transepithelial electrical resistance (TEER)	CYN 1–10 µM/3–24 h	Immortalized human colorectal adenocarcinoma cell line	16.7–20.5% intestinal permeability in 24 h; epithelial integrity not significantly altered		[30]
CaCo-2	Permeability of pseudoepithelial layer	CYN 1.9–48 µM/24–48 h	Immortalized human colorectal adenocarcinoma cell line	Apparent permeability: 3.45 × 10^−7^ cm/s (absorptive), 6.41 × 10^−7^ cm/s (secretive); epithelial permeability (increase): Tenfold (absorptive), 0.7-fold (secretive);		[31]
CaCo-2, NCI-87, HCT-8, HuTu-80, Vero, C3A, HepG2	MTT assay for cell viability	CYN 0.25–5 µM/1–7 days	Gastro-intestinal and hepatic cell lines	CYN sensitivity decreased in cell lines as follows: Gastric > duodenal > ileal > colonic; EC_50_ is 6.5 ± 3.3 µM for CaCo-2		[32]
CaCo-2, HepaRG	Cytokinesis-block micronucleus assay	CYN	The same human hepatocyte cell line	CYN increased the frequency of micronuclei in binucleated cells	Cytochrome P450	[33]
CaCo-2	Bradford assay for total protein content, MTS reduction for cell viability, GSH and ROS content, electron microscopy	CYN 0.7–96 µM/24–48 h	The same	Lipid degeneration, mitochondrial damage, nucleolar segregation with altered nuclei, ultrastructur	Membranes, mitochondria, nuclei, endosomes	[34]
HIEC-6	MTT assay	CYN 1.0–11 µM/24 h	Human intestinal epithelial cell line	Reduced cell viability by 13.4% and 21.8%		[35]
mES	Real-time PCR (RT-PCR)	CYN 0–1 *μ*g/mL/24–168 h	Undifferentiated mouse embryonic stem cell	EC_50_ 0.86 *μ*g/mL/24 h, LOEC is 1 *μ*g/mL	Oct4 Brachyury Nestin	Reference [36]
HepG2	MTS test, flow cytometry, RT-PCR	0.125, 0.25, 0.5 µg/mL CYN + MC-LR, 1 µg/mL/24 and 72 h	Human hepatocellular carcinoma cell line	DNA double-strand breaks after 72 h, upregulation of CYP1A1 by CYN and CYN + MC-LR via CDKN1A and GADD45 A genes, cells arrested in G0G0/*G*1 phase	DNA	[37]
Rat hepatocytes	LDH leakage; cysteine, ATP, and GSH assay	CYN 2.5–5 µM/12 h	Rat hepatocyte cell culture	Inhibition of GSH synthesis	GSH, cytochrome P450	[38]
Mouse hepatocytes	LDH leakage, protein synthesis	CYN 2.5–5 µM/4–18 h	Mouse hepatocyte cell culture	Inhibition of LDH leakage, max at 0.5 µM CYN; CYN, 1–5 µM lead to 52%–82% cell death	Protein synthesis, cytochrome P450	[39]
HepG2	MTS assay, live/dead staining, qPCR, flow cytometry, confocal z-stack imaging	CYN 0.125, 0.25, 0.5 *μ*g/mL/72 h	Human hepatocellular carcinoma cell line	CYN deregulated genes for phase I and II enzymes, for cell proliferation; apoptosis and DNA damage response	DNA, expression of many enzymes	[40]
WIL2-NS	Centromere staining, PCR, cytokinesis-block micronucleus assay	CYN 1, 3, 6, 10 *μ*g/mL/24 h	Lymphoblastoid cell-line	Cytogenetic damage by DNA- and kinetochore/spindle-dependent mechanisms	Centromere, micronuclei	[41]
HepG2	LDH leakage, MTT assay, flow cytometry, immunocytochemical staining	CYN 0.1–0.5 µg/mL/24–96 h	Human hepatoma cells	Genotoxic effect by DNA double-strand breaks	DNA	[42]
CLC		CYN 0.1, 0.5, 1 µg/mL/24 h	Common carp (*Cyprinus carpio* L.) leucocyte cell line	Decreased cell membrane integrity, GSH/GSSG ratio, inhibited cell proliferation, DNA damage, increased ROS and ATP levels (1 µg/mL)	Micronuclei, GSH, ATP, SOD	[43]
HepG2	MTS assay, qPCR, flow cytometry	CYN 0.5 *μ*g/mL/24 or 72 h, biphenols 10 *μ*g/mL	Human hepatoma cells	Deregulation of some genes was more pronounced after exposure to the mixture	DNA	[44]
CaCo-2			Immortalized human colorectal adenocarcinoma cell line	Apparent permeability of the pseudoepithelial cell layer to MC-LR		[45]
A7r5	AO/EB staining assay and comet assay, flow cytometry, qRT-PCR	CYN 20, 200, 2000 nM/24 h	Rat vascular smooth muscle cells	CYN induced apoptosis in a dose-dependent manner, DNA damage	Actin, p53, Bax/Bcl2, SOD, CAT and GPX	[46]
LLC-PK1	Flow cytometry, qRT-PCR	1.0 *μ*g/mL	Renal epithelial cells derived from proximal tubules	CYN induced necrosis and increased gene expression of Na+/K + –Atpase	Na^+^/K^+^-ATPase activity	[47]
Human keratinocytes	LDH leakage, WST-1 cell proliferation assay, Scratch test, crystal violet assay	1, 10 *μ*g/mL for 24/48h	Primary human keratinocytes	CYN induced cytotoxicity, impaired migration, and inhibition of proliferation		[48]

Abbreviations: AO/EB staining – acridine orange/ethidium bromide staining; ATP – adenosine triphosphate; CAT – catalase; GPX – glutathione peroxidase; GSH – glutathione; MTS – 3-(4,5-dimethylthiazol-2-yl)-2,5-diphenyl-2H-tetrazolium bromide; RP-PCR–reverse transcription polymerase chain reaction; RT-qPCR – quantitative reverse transcription polymerase chain reaction; SOD – superoxide dismutase.

**Table 5:** Cytotoxicity of depsipeptides.

Cell type	Assay	Conditions	Tissue of origin	Main effects	Targets	Ref.
N2a, NCI H-460	MTT assay	Pal A IC_50_: 17.2 *μ*M/24 h; 39.7 *μ*M/48 h	Neuro2a mouse neuroblastoma cells; human lung carcinoma cells	Blockage of the voltage-gated sodium channel, modest cytotoxic effects.	Voltage-gated sodium channel	[51]
NCI H-460	MTT reduction	20 *μ*g/well	Human lung carcinoma cells	Cytotoxicity, IC_50_: 2.6 nM/48 h	G1-phase cell cycle arrest, apoptosis	[52]
60 cancer cell lines	Flow cytometry		Human cells from lung, colon, leukemia, melanoma, CNS, ovarian, prostate, breast and renal cancers	Cytostatic and cytotoxic effects – increase the number of cells in G_1_, little change in G_2_/M and loss of cells in S-phase. GI_50_ for CoA: 2.8 nM to MDA-MB-231 7.4 nM to LOX IMVI 7.4 nM to HL-60(TB)	Novel unknown mechanism; no effect on tubulin or actin in cytoskeletal assays	[53]
MDCK cells infected with influenza virus A/WSN/33/London (H1N1)	Dye uptake assay using neutral red	Ich A and B in nontoxic conc. 12.5–100 *μ*g/mL/30 min.	Canine kidney	Antiviral activity, IC50: 12.5 *μ*g/mL	Non-trypsin protease inhibition	[54]
60 human cancer cell lines (NCI-60 cell lines)	Biokinetics reader, fluorescence detection, acute toxicity determination, MTT assay, hollow fiber assay		Human leukemia, melanoma, lung, colon, CNS, ovarian, prostate, breast and renal cancer cell lines	Antitumor and antifungal activities; GI_50_ for NCI-H322M: KF2–0.131 *μ*M; KF4–0.133 *μ*M; KF–0.191 *μ*M; GI_50_ = 0.123 *μ*M for human prostate (DU-145); GI_50_ = 0.453 *μ*M for breast cancer (HS 578T) cell lines. IC_50_ for *C. neoforman*s: KF–1.53 *μ*M, KF2–0.95 *μ*M	—	[55]
WHCO1, WHCO6, ME180	MTT assay		WHCO1,06–esophageal and ME180–cervical cancer cells	Cytotoxicity: IC_50_ for HD16 WHCO1–4.3 *μ*g/mL; WHCO6–10.1 *μ*g/mL; ME180–8.3 *μ*g/mL	—	[56]
P388, A549, PC3, HCT8, SK-OV	MTT assay, scintillation counting		P388-murine leukemia, A549-lung carcinoma, PC3-prostate cancer, HCT8 -ileocecal colorectal adenocarcinoma and SK-OV-ovarian cancer cells	Cytotoxicity and antimalarial activity; IC_50_ for cancer lines: P388–24.4 nM; A549–2.4 nM; PC3–2.6 nM; HCT8–2.1 nM; SK-OV–4.5 nM; IC_50_ for *Plas. Falciparum*–0.29 *μ*M	Mitochondria-induced apoptosis, lag C selectively binding to the prohibitin	[57]
HT-29, MCF7	MTT assay, disc diffusion assay		HT-29 colon adenocarcinoma, MCF7 breast cancer cells	Cytotoxicity and antimycobacterial activity against *M. tuberculosis.* For HT-29 IC_50_: Pit A–13 *μ*M; Pit B–13 *μ*M; Pit C–67 *μ*M; Pit *D* – >100 *μ*M; Pit E–75 *μ*M; Pit F–87 *μ*M and for MCF7 IC_50_ : Pit A–13 *μ*M; Pit B–11 *μ*M; Pit C–73 *μ*M; Pit *D* – >100 *μ*M; Pit E – >100 *μ*M; Pit F–83 *μ*M	-	[58]
HeLa cells	WST-1 assay, Immuno-precipitation	100 nM aurilide	Human cervical cancer cells	Cytotoxicity, mitochondria-induced apoptosis	Prohibitin 1, optic atrophy 1	[59]
NCI–H460, neuro-2a	MTT reduction.		NCI–H460 – human lung tumor, neuro-2a – mouse neuroblastoma cell lines	Cytotoxicity for NCI–H460 LC_50_ is: Wew A–0.65 *μ*M; wew B–0.43 *μ*M; wew C–5.9 *μ*M; wew D–3.5 *μ*M; for neuro-2a LC_50_: Wew A – 0.49 *μ*M; wew B–0.20 *μ*M; wew C–10.7 *μ*M; wew D–1.9 *μ*M		[60]
60 human cancer cell lines (NCI-60 cell lines), MEFs	Immunoblot, MTT assay, Trypan blue exclusion, LDH assay, caspase activity assay, autophagy assays, EGF receptor degradation assays		Human cancer cells from leukemia, melanoma, lung, colon, CNS, ovarian, prostate, breast, renal cancers	Cytotoxicity, apoptosis, and inhibition of cell growth. EC_50_ cytotoxicity is < 100 nM for human U87-MG and SF-295 cells, and for mouse embryonic fibroblasts	Caspase-3, extensive cytoplasmic vacuolization, mTor-independent pathway	[61]
HCC2218, UACC-893, T-47D and >50 others	Growth inhibition assay, immune-precipitation study, SEAP secretion assay		Human breast, ovarian, endometrial, pancreatic, skin, lung, and colon cancer cell lines; rat pancreatic exocrine cell line	Cytotoxicity, blocking of cotranslational translocation. IC_50_ = 5–50 nM for different cell types	Sec61 in the ER membrane.	[62]

Abbreviations: EGF – epidermal growth factor; SEAP – secreted embryonic alkaline phosphatase.

**Table 6:** Cytotoxicity of LPS.

Cell type	Assay	Conditions	Tissue of origin	Main effects	Ref.
Microglia	Superoxide anion (O_2_^−^) generation, cell viability by LDH release, thromboxane B_2_ (TXB_2_), immunoassay, gelatinase zymography for matrix metalloproteinase-2 (MMP-2), and matrix metalloproteinase-9 (MMP-9), rat-specific ELISA for cytokines and chemokines	*Microcystis aeruginosa* LPS strain UTCC 299; 0.1–100,000 ng/mL/17 h *E. coli* LPS (0.1–100 ng/mL) as control	Rat neonatal brain microglia	Enhanced O_2_^−^ generation, limited inflammatory mediator generation; MMP-9, macrophage inflammatory protein-2 (MIP-2/CXCL2) release, TXB_2_, concurrent with maximal O_2_^−^ generation; elevated TXB_2_, MMP-9, tumor necrosis factor *α* (TNF-*α*), interleukin 1-*α* (IL-1*α*), and interleukin-6 (IL-6), macrophage inflammatory protein 1*α* (MIP-1*α*/CCL3), and MIP-2/CXCL2; LPS activates brain microglia *in vitro* and the release of O_2_^−^, inflammatory mediators	[64]
Microglia		0.1–100 000 ng/mL Oscillatoria sp. LPS; 17 h	Rat neonatal microglia	Classical and alternative activation; pro-inflammatory and anti-inflammatory mediator release	[65]
Microglia		*Scytonema javanicum* and *S. ocellatum* LPS	Rat neonatal microglia	Concentration-dependent O₂^−^, MMP-9, IL-6 TNF-*α*, MIP-2/CXCL-2, CINC-1/CXCL-1, MIP-1*α*/CCL3, IL-10 release	[66]
Meningioma cells and meningioma–primary human macrophage	Sandwich immunoassay	Cyanobacterial LPS antagonist (CyP) 1–20 *μ*g/monolayer	Human meningioma cells and meningioma–primary human macrophage co-cultures	Cyanobacterial LPS inhibits cytokine production and augments the anti-inflammatory response when combined with benzylpenicillin	[67]
Microglia	Immunocytochemical and immunofluorescent assay, ELISA, immunoblotting, live-cell imaging analyses	Cyanobacteria-derived TLR4 antagonist—a highly (95%) purified form of LPS-like molecule from Oscillatoria planktothrix sp. 20 *μ*g/mL for 24 h,	Primary cultures from mouse spinal cords	TLR4 antagonists could be considered as a candidate of protective agents for motor neurons in degenerative diseases	[68]
Spleen cells		Hot-water extract of *Spirulina platensis*	*In vitro* cultures of murine spleen and thymus cells	Increased proliferation of spleen cells; enhanced IL-1 production from peritoneal macrophages	[69]
hTHP-1	ELISA, real-time PCR	Cyanobacterial LPS antagonist (CyP) from *Oscillatoria planktothrix FP1*; 10 µg/mL/5 h	Human THP-1 monocytic cell line	CyP is able to induce cross-tolerance to *E*. *coli* LPS by inhibiting TNF-*α* production	[70]

**Table 7:** Cytotoxicity of lyngbyatoxins.

Cell type	Assay	Conditions	Tissue of origin	Main effects	Targets	Ref.
Fibroblasts	MTT assay, [^3^H]-thymidine incorporation assay	15 mg/mL (w/v) of the cyanobacterial extract/4 h or 24h for MTT test; 24 h for [^3^H]-thymidine incorporation assay	Primary mouse thymus fibroblasts	80% inhibition of cell proliferation, morphology and attachment in 24 h	DNA, cell membrane, cytoskeleton	[70]
FL			Normal amniotic cells, human	Stimulation of MTT reduction after 4 *h* > 40% *vs* control cells; decreased cell viability to 32% of controls in 24 h		
A2058			Human metastatic melanoma	Cytotoxic in 24 h		
RD			Human embryonic myosarcoma	Cytotoxic in 24 h		
3T3			Mouse embryonic fibroblasts	92% inhibition of cell proliferation		
L1210	MTT	Lyngbyatoxin A and 12-epi-lyngbyatoxin A/18 h	Mouse lymphocytic leukemia cell line	Cytotoxic effect; IC_50_ = 8.1 *μ*M *lyngbyatoxin A*; IC_50_ = 20.4 *μ*M *12-epi-lyngbyatoxin A*	PKC isozymes	[71]
HL-60 C	Test of induction of cell adhesion	Lyngbyatoxin A and debromoaplysia toxin/48 h	Human promyelocytic leukemia cells	For 50% cell adhesion to the flasks—7 ng/mL *Lyngbyatoxin A* and 700 ng/mL *debromoaplysiatoxin*	Cell membrane	[72]
DS 19	Test of inhibition of terminal differentiation		Mouse erythroleukemia cells transformed by Friend leukemia virus strain 745A	Inhibition of terminal differentiation in 50% of the cells with 0.35 ng/mL *Lyngbyatoxin A* and 150 ng/mL *debromoaplysiatoxin*		
Neuro-2a	MTT	24 h	Mouse neuroblastoma cells	Cytotoxicity IC_50_ = 2.2 *μ*M of hermitamides A; IC_50_ = 5.5 *μ*M hermitamides B		[73]
CHO	Patch-clamp	0.1–30 *μ*M neo-debromoaplysia toxin G and H	Chinese hamster ovary cells	Potassium channel Kv1.5 block; IC_50_ = 1.79 *μ*M debromo aplysiatoxin G and IC_50_ = 1.46 *μ*M debromoaplysia toxin H	Voltage-gated potassium channels Kv1.5 (KCNA5)	[74]

**Table 8:** Cytotoxicity of MC.

Cell type	Assay	Conditions	Tissue of origin	Main effects	Targets	Ref.
CaCo-2	Immuno-localization of MC uptake	MC-LR 1–75 µM/30 min–24 h	Immortalized human colorectal adenocarcinoma cell line	Artificial epithelial cell layer is highly permeable to MC-LR		[45]
CaCo-2	Gene expression, transcriptomics	MC-LR 10–100 µM/4–24 h	The same	Oxidative stress	ERK/MAPK and cell cycle pathway molecules	[75]
CaCo-2	Comet assay, MTT assay (for viability)	MC-LR 0.2–10 µM/4–48 h	The same	20% damaged DNA after 0.2 µM/4 h MC-LR; 40% reduced cell viability after MC-LR 10 µM/48 h,	DNA	[76]
CaCo-2	Protein phosphatase (PP) inhibition, LDH leakage, cell morphology and proliferation	1–50 µM MC-LR, -LF and -LW for 22–48 h	The same	PP inhibition—3.0 nM MC-LF, 3.8 nM MC-LW, 1.0 nM MC-LR, EC_50_ of LDH leakage: 25% (50 µM MC-LR), 36% (MC-LW), 51% (MC-LF), chromatin cell shrinkage, condensation, membrane blebbing, and cytoskeletal reorganization	PP, cell membrane, chromatin, cytoskeleton	[77]
CaCo-2	Bradford assay, MTS reduction (for viability), neutral red uptake	MC-LR, –RR and -YR, 50–200 µM/24–48 h	The same	EC_50_ reduction of total protein content by MC-LR 111.1 ± 3 µM/24 h and MC-RR ˃200 µM/48 h; neutral red uptake—MC-YR 57.3 µM/48h	Protein synthesis	[78, 79]
CaCo-2	Immuno-localization of microcystins	MC-LR, –RR, 1–50 µM/30 min–24 h	The same	Facilitated MC uptake in <1 h by organic anion transporters, active excretion	Organic anion transporters 3A1 and 4A1	[80]
HIEC-6	Cell counting Kit-8 for viability, western blot, TEER, PP2A activity	MC-LR 0–50 µM/6–24 h	Human intestinal (colon) epithelial cell line	Viability–12.5 µM/24 h; TEER at 50 µM/12 h and at 12.5 µM/24 h; apoptosis at 12.5 µM/24 h; western blot at 12.5 µM/24 h; occludin; claudin not affected), 25 µM/24 h; ZO-1; PP2A activity decreases from 12.5 µM/24 h	PP2A, occludin, claudin	[81]
HEK293	Western blot, luciferase assay, rtPCR	MC-LR 10 µM/24 h	Human embryonic kidney cells	PP2A inhibition, enhanced proto-oncogene C-myc expression	PP2A, c-Myc protein, proto-oncogene C-myc	[82]
NCC	PP2A, PP2B, PP2C activity, western blot, Akt, p38, JNK, PI3K assays, genechip analyses;	MC-LR, 0.0001–1.0 µg/24 h	Immortalized colorectal crypt cells	Constitute activation of Akt/*p*38 and JNK/MAPK pathways	Akt, p38, JNK	[83]
HBE1, 16HBE14o-	RT-PCR, western blot, RTCA, neutral red uptake	MC-LR 1–20 µM/48 h	Human bronchial epithelial cell lines	No effect on viability, ERK1/2 and p38 activities were not changed	ERK1/2 and p38 not influenced	[9]
DLD-1, HT-29	Western blot, RT-qPCR, knockdown of SMAD2 by siRNA, migration and invasion assay	MC-LR, 0.1–50 nM/24 h	Human colorectal cancer cells	Induction of SMAD2 signal transducer and transcriptional modulating protein expression, its activating phosphorylation by PI3K/Akt, increased migration (epithelial-mesenchymal transition of both cell types)	PI3K/Akt, SMAD2,	[84]
BALB/c	mRNA	MC-LR 1–1000 nmol/L/6 h	Mouse peritoneal macrophages	Decreased transcription of mRNA for iNOS, IL‐1*β*, TNF‐*α*, GM‐CSF, and IFN‐*γ*; reduced inflammatory response to LPS	iNOS, IL‐1*β*, TNF‐*α*, GM‐CSF and IFN‐*γ*	[85]
RAW 264.7 macrophages	Western blot, ELISA	MC-LR, 1–1000 nmol/L/30 min–24 h	Abelson leukemia virus-transformed cell line from BALB/c mice	Activation of NF-*κ*B with 1000 nM and ERK1/2 with 100 nM; TNF-*α* synthesis (1 nM)	NF-*κ*B, ERK1/2, TNF-*α*	[86]
HepG2	RT-qPCR, Western blot, MTT assay, mitochondrial membrane potential (MMP)	MC-LR, 0.01–5 µM/3, 6, 12 and 24 h	Human hepatocellular carcinoma cell line	MMP loss, SOD induction in hypoxia, inhibitory apoptosis protein (c-IAP2) up-regulated in normoxic condition	Mitochondrial dehydrogenase, SOD, c-IAP2	[87]
A549	MTT assay, PP2A activity, Western blot, proliferation	MC-LR, 0.5–10 µM/24 h	Human non-small-cells lung cancer cells	Rearrangements of filamentous actin and microtubules due to PP2A/C (>1 µM) and p38 MAPK activation (0.5–10 µM); p-Blc-2, p-Bad (1.0–10 µM)	Microtubules and filamentous actin (cytoskeleton), PP2A/C, p38,	[88]
HEK293	Western blot, cell detachment, PP2A activity, MTT assay	MC-LR, 0.5–10 µM/24 h	Human embryonic kidney cells	PP2A inhibition (>5 µM); PP2A activation (1–2 µM); cell anoikis	PP2A catalytical and regulatory subunits	[89]
PC12	Western blot, PP2A activity, immuno-fluorescence	MC-LR, 0.1–10 µM/6 h	Pheochromocytoma cells of the rat adrenal medulla	Rearrangement of filamentous actin and microtubules due to PP2A (>0.5 µM) and p38 MAPK	PP2A, p38 MAPK, HSP27	[90]
HL7702	PP2A activity, western blot, immuno-fluorescence	MC-LR, 5 or 10 µM for 30 min to 24 h;	Human normal liver cell line	Activation of p38 MAPK, JNK and ERK1/2, HSP27-sensiitive cytoskeleton reassembly, PP2A inhibition in 6–24 h; activated phosphorylation of tau (by P38 MAPK) and VASP	p38 MAPK, JNK, ERK1/2, PP2A; tau and VASP components of cytoskeleton	[91, 92]
SMMC-7721	PP2A activity, western blot, PKA activity and Rac1/Cdc42 activity immuno-fluorescence, immuno-precipitation	MC-LR, 0.5–10 µM/24 h	Human liver cancer cell line	p-HSP27, p-VASP and p-cofilin contributed to cytoskeleton change; PP2A inhibition (>0.5 µM); disorder of cytoskeleton	HSP27, VASP, cofilin, PKA, Rac1, PP2A	[93]
HepaRG	Cytopathic effects, RNA quantified by Agilent RNA 6000 Nano kit	MC-LR, –RR 10, 100 and 1000 ng/2 h	Human hepatocyte cell line	Increase of RNA of apoptotic and inflammatory gene; many cellular pathways activated		[94]
HL7702	Real-time cell analyzer (RTCA) proliferation, cell cycle analysis, western blot, PP2A activity, MTT assay, immuno-fluorescence	MC-LR, 1, 5, 10 µM/1–96 h	Human normal liver cell line	MC-LR promoted HL7702 cell proliferation (36–48 h); activation of Akt/S6K1 cascade; PP2A activity (>1 µM), hyper-phosphorylation of Bcl-2, Bad, c-Myc and c-Jun, 1–10 µM	PI3K/Akt/S6K1, hyper-phosphorylation of Bcl-2, Bad, c-Myc and c-Jun	[95]
HBE	MTT and Annexin V/PI assay, ROS and MMP measurements, western blot	MC-LR, 1, 10, 20, 30, 40 µg/mL/24, 48 h	Human bronchial epithelial cells	Inducing mitochondria-dependent apoptosis (1–40 µg/mL), MMP decreases at 10 µg/mL	Caspases	[89]
Huh7		MC-LR, 0.5–50 *μ*M/6–72 h	Human hepatoma cells	5 *μ*M MC-LR induced PP2A mRNA expression, p-CREB, expression of NF-*κ*B, IFN-*α*, and several INF*α*-stimulated genes are activated	NF-*κ*B, p-CREB, DNA	[96]

Abbreviations: CREB – cAMP responsive element-binding protein; ERK/MAPK – extracellular signal-regulated kinase/mitogen-activated protein kinase; GM‐CSF – granulocyte macrophage colony-stimulating factor; IFN-*γ* – interferon gamma; iNOS – inducible nitric oxide synthase; JNK–c – Jun N-terminal kinases; mRNA – messenger RNA; siRNA – small interfering RNA, VASP – vasodilator-stimulated phosphoprotein.

**Table 9:** Cytotoxicity of nodularins.

Cell type	Assay	Conditions	Tissue of origin	Main effects	Targets	Ref.
CLC	Fluorometric cell membrane integrity, cell viability and ROS measurements, caspase-glo 3/7 assay, ELISA	NOD, 0.001, 0.01, 0.05, 0.1 *μ*g/mL/24 h	Carp leukocyte cell line and head kidney leukocytes	Cell viability, membrane integrity at 0.1 *μ*g/mL, DNA fragmentation and caspases 3/7 activation at > 0.1 *μ*g/mL, ROS increase in 60 min in >0.01 *μ*g/mL, GSH decrease at >0.001/24 h	GSH/GSSG, DNA, membranes, caspases	[98]
CLC and kidney leucocytes	Fluorometric cell viability, ROS and nitrogen species (NS) measurements	0.001, 0.01, 0.05, or 0.1 *μ*g/mL/24 h	Carp leukocyte cell line, kidney leukocytes	Cytotoxicity ≥0.05 *μ*g/mL, ROS and NS increase, expression of TNF-*α*, IL-10, less TGF-*β*	DNA expression	[99]
HepG2	Micronucleus assay, Flow cytometry, comet assay, DNA damage	NOD, 1–10 *μ*g/mL, for 6, 12, 24, 48 h	Human hepatoma cell line	DNA damage >1 *μ*g/mL, apoptosis from1 *μ*g/mL/48 h	DNA, cellular and mitochondrial membranes	[100]
HepG2	RT-PCR, siRNA, flow cytometry, transfection of NF-*κ*B immunobloting	NOD, 2.5, 5, 7.5, 10 *μ*M/24 h	Human hepatoma cell line	Induces fas receptor (fas) and fas ligand (FasL) expression and apoptosis	NF-*κ*B pathway, fas, FasL	[101]
HepG2 and Huh7	ATF-6 activity qPCR, TNF-*α* ELISA, immunoblotting	NOD, 0.1, 1, 5 *μ*M for 24, 48 and 72 h	Human hepatoma cell lines	Induction of TNF-*α* protein, CAAT/enhancer-binding protein-homologous,	TNF-*α*, ERK 1/2 MAPK, ER, IL-8, CHOP ER-stress—2.5 nM	[102]
HepG2	qPCR, MTT assay, comet assay, cytokinesis micronucleus assay	NOD, 0.01, 0.1 and 1 *μ*g/mL	Human hepatoma cell line	DNA damage; apoptosis (BAX, BCL2) genes, ROS increase, oxidative stress	DNA, ROS	[103]

**Table 10:** Cytotoxicity of cyanobacterial retinoids.

Cell type	Assay	Conditions	Tissue of origin	Main effects	Targets	Ref.
P19/A15	Bioluminescence reporter assay; calcein AM cell viability assay	0.25, 0.5, 1 and 2 g dm/l/24 h; water extracts 2.5x–20x environmental water/24h	Murine embryonal carcinoma cells stably transfected with firefly luciferase gene	Retinoid-like activity, max 263 ng retinoid eq/L; cytotoxic effect at 20x	RAR	[104]
P19/A15	Bioluminescence reporter assay;	0.25, 0.5, 1 and 2 g dw/l/24h	Murine embryonal carcinoma cells	Retinoid-like activity	RAR	[105]
P19/A15	RAR/RXR transactivation assay	Cyanobacterial extracts 0.125–2 g dw/l/24 h	Murine embryonal carcinoma cells	Retinoid acid receptor (RAR) activity	RAR	[106]
P19/A15	Bioluminescence reporter assay	Cyanobacterial extracts 0.25–2 g dm/l and exudates 2.5×–20×/24 h	Murine embryonal carcinoma cells	Retinoid acid receptor activity	RAR	[107]
P19/A15	Bioluminescence reporter assay	1x–20x concentrated cyanobacterial and algal exudates/24h	Murine embryonal carcinoma cells	Retinoid-like activity	RAR	[108]
HepG2	MTT, comet assay, cytokinesis-block micronucleus (cytome) assay	0.04–2 mg/mL/24h for MTT, 0.2 mg dm/mL/24h for cytokinesis-block micronucleus assay	Human hepatocellular carcinoma cells	Significant genotoxic effects of retinoic acid from the extracts	DNA	[109]

**Table 11:** Cytotoxicity of SXT.

Cell type	Assay	Conditions	Tissue of origin	Main effects	Ref.
Neuro-2A	HPLC, LC-MS/MS, Jellett rapid test, MTT assay	STX, 0.05–200 ng/mL	Mouse neuroblastoma cell line	Screening assay for determination of toxicity and comparison of various methods for detection of toxins	[111]
IEC-6, Caco-2	HPLC,	Gonyautoxin, 100 *μ*M for 1–60 min	Human colorectal, adenocarcinoma cell line	IEC-6 cells secrete the toxin, Caco-2 cells absorb it Na^+^-dependently	[112]
